# Feasibility of a placebo-controlled trial of antibiotics for possible urinary tract infection in care homes: a qualitative interview study

**DOI:** 10.3399/BJGPO.2023.0014

**Published:** 2023-08-09

**Authors:** Christopher R Wilcox, Louise Worswick, Ingrid Muller, Abigail Moore, Gail Hayward, Mark Lown, Michael Moore, Paul Little, Nick Francis

**Affiliations:** 1 Primary Care and Population Sciences, Faculty of Medicine, University of Southampton, Southampton, UK; 2 Primary Care Research Centre, Faculty of Medicine, University of Southampton, Southampton, UK; 3 Nuffield Department of Primary Care Health Sciences, University of Oxford, Oxford, UK

**Keywords:** urinary tract infections, qualitative research, primary health care, general practice, care home, feasibility studies

## Abstract

**Background:**

Diagnosis of suspected urinary tract infection (UTI) in care and nursing home residents is commonly based on vague non-localising symptoms (for example, confusion), potentially leading to inappropriate antibiotic prescription. The safety of withholding antibiotics in such cases could be addressed by a randomised controlled trial (RCT); however, this would require close monitoring of residents, and support from care home staff, clinicians, residents, and families.

**Aim:**

To explore the views of residential care and nursing home staff (herein referred to as care home staff) and primary care clinicians on the feasibility and design of a potential RCT of antibiotics for suspected UTI in care home residents, with no localising urinary symptoms.

**Design & setting:**

A qualitative interview study with primary care clinicians and care home staff in the UK.

**Method:**

Semi-structured interviews with 16 care home staff and 11 primary care clinicians were thematically analysed.

**Results:**

Participants were broadly supportive of the proposed RCT. The safety of residents was a priority and there was strong support for using the RESTORE2 (Recognise Early Soft Signs, Take Observations, Respond, Escalate) assessment tool to monitor residents; however, there were concerns about associated training requirements, especially for night and temporary staff. Effective communication (with residents, families, and staff) was deemed essential, and carers were confident that residents and families would be supportive of the RCT if the rationale was clearly explained and safety systems were robust. There were mixed views on a placebo-controlled design. The perceived additional burden was seen as a potential barrier, and the use of temporary staff and the out-of-hours period were highlighted as potential risk areas.

**Conclusion:**

The support for this potential trial was encouraging. Future development will need to prioritise resident safety (especially in the out-of-hours period), effective communication, and minimising additional burden on staff to optimise recruitment.

## How this fits in

Diagnosis of UTIs in care homes is commonly based on vague symptoms (for example, confusion), leading to potentially inappropriate antibiotic prescription. This qualitative interview study with care home staff and primary care clinicians demonstrated support for a future randomised trial assessing the safety of withholding antibiotics in such cases. Future trial development will need to prioritise resident safety (especially in the out-of-hours period), effective communication, and minimising additional burden on staff.

## Introduction

There are increasing concerns about antibiotic overuse in the care home population, particularly for suspected UTIs.^1^ Overuse drives antimicrobial resistance,^2^ and increases adverse effects and healthcare costs.^3,4^ Accurate diagnosis of UTI in care home residents is challenging. While some have localising urinary symptoms (such as dysuria), non-specific symptoms (such as confusion) are the most common reason for suspecting a UTI, despite a myriad of other possible causes.^5,6^ This can lead to potentially inappropriate antibiotic treatment.^1^ The diagnostic challenge is compounded by physical and cognitive impairments, as well as the high prevalence of asymptomatic bacteriuria (up to 50%).^7^ However, undertreatment may lead to severe infection, so accurate diagnosis is key.

Evidence for the link between non-specific symptoms (especially confusion) and UTI remains unclear,^1^ and a recent qualitative study highlighted a desire among clinicians for research that explores effective management strategies (including the safety of withholding of antibiotics).^8^ A high-quality, randomised, placebo-controlled trial of antibiotics for suspected UTI in care home residents who present with non-specific symptoms alone might help address this uncertainty, and potentially give clinicians confidence to withhold antibiotics.^5,8^

Given the vulnerability of this population, such a study would require clear inclusion and exclusion criteria to exclude those deemed to be more severely unwell or at high risk of deterioration, as well as a safe process for monitoring participants. This could include ‘early warning scores’, such as National Early Warning Score 2 (NEWS2) (based on physical observations) or RESTORE2 (a tool developed for care homes that combines NEWS2 with an element of clinical judgement or ‘soft signs’), to monitor residents and guide clinical escalation.^9,10^

The use of early warning scores significantly increased across UK care homes during the COVID-19 pandemic;^11,12^ however, concerns have been raised over their use outside the hospital setting in which NEWS2 was developed.^13^ Support from care home staff is also essential for successful recruitment into trials in this setting,^14,15^ but while staff play a key role in the assessment and management of suspected UTIs, they may have limited awareness of other causes of non-specific symptoms or functional decline,^16,17^ and the potential harms of inappropriate antibiotic prescribing.^18^

The aim of this qualitative interview study was to explore the views of care home staff and primary care clinicians on the following: 1) the acceptability of conducting a trial of antibiotics in care and nursing home residents with ‘suspected UTI’; 2) the selection of participants and the use of or early warning scores to improve safety for trial participants; and 3) the design and potential barriers and facilitators to conducting such a trial.

## Method

### Study design

This was a qualitative interview study with primary care clinicians and care home staff. An exploratory literature review was conducted in September 2021 to inform the development of the study protocol and interview topic guides (see Supplementary Information S1 and S2). Semi-structured interviews were undertaken from March–June 2022. Stakeholder meetings with care and nursing home residents, their families, and staff, took place in September and October 2022.

### Context

English residential care homes are staffed 24 hours a day by care workers without nursing qualifications, and support residents with personal care. Nursing homes are staffed by care workers and registered nursing staff, for residents requiring additonal nursing care.^19^ The proportion of care and nursing staff will vary, and both residential care and nursing homes may also employ temporary staff. Quality assurance will be overseen nationally by the Care Quality Commission. For clinical care, homes are registered with a GP practice, and GPs and/or other clinical staff (including nurse practitioners and paramedics) will provide clinical care to residents when staff raise concerns. Some may have access to additional clinical services, including ‘telemedicine’ and frailty teams (usually staffed by nurse practitioners and paramedics), who may provide clinical advice, support, and assessment alongside the registered GP practice.

### Recruitment

Care home staff were eligible if they: 1) had >6 months' experience; and 2) were involved in decisions about the management of residents with suspected UTI. Primary care clinicians (including GPs, advanced nurse practitioners, and paramedics) were eligible if they: 1) had a clinical role in any setting; and 2) had experience of assessing care home residents with suspected UTI. A purposive sampling approach was taken to identify participants from different roles and seniority grades, with assistance from the Clinical Research Network Wessex. Participants were offered a £20 voucher. Participants were asked to invite colleagues (snowball sampling), and the study was advertised on social media.

### Data collection

Semi-structured interviews^20^ were conducted through video call with a qualitative researcher using interview topic guides (see Supplementary Information S1 and S2), which were refined after initial piloting. The interview guide included questions on participants’ experience of managing residents with suspected UTI, experience and views on early warning scores, and their views on a potential future RCT of antibiotics for possible UTI. A hypothetical ‘trial outline’ was shared with participants (see Supplementary Information S3 and S4). Interviews were audiorecorded and transcribed. Recruitment ended once data saturation was reached (when new categories or themes stopped emerging from the data).

### Data analysis

Thematic analysis was undertaken using an inductive approach.^21,22^ One author initially gained familiarisation with transcripts and coded the narrative into units of meaning. Emerging codes were scrutinised for patterns, similarities, differences, contradictions, and observations, which led to groups of codes and themes being generated. A coding framework was developed by placing each item of coded data in a named category in the framework. Initial codes and themes were discussed with the study team and refined. Data were reported in compliance with the COnsolidated criteria for REporting Qualitative research (COREQ) checklist.^23^

### Patient and public involvement

Two patient and public involvement (PPI) members (a care home worker and a relative of a care home resident) attended study meetings. They assisted with recruitment, drafting of study documents including topic guides, write-up, stakeholder meetings, data interpretation, and dissemination of findings.

## Results

### Participants

Sixteen care home staff (Table 1) and 11 primary care clinicians participated (Table 2). A broad spectrum of participants were recruited in relation to age, sex, role, seniority, and experience.

**Table 1.** Care home staff demographics

Participant ID	Home type	Main role	Time working in present care home	Total time working in care homes	Previous experience of research	Age, years	Sex
VENCH016	Residential	Deputy manager	8 years	41 years	Yes	57	F
VENCH017	Residential dementia	Registered manager	13 years	45 years	Yes	62	F
VENCH018	Dual registered	Night-care coordinator	20 years	30 years	No	53	F
VENCH019	Residential	Registered manager	5 months	15 years	No	45	F
VENCH020	Residential	Head of care	7 years	10 years	No	34	F
VENCH021	Residential	Senior manager	27 years	27 years	No	70	M
VENCH022	Residential LD and nursing	Senior nurse	4 years	10 years	No	45	F
VENCH023	Residential LD and nursing	Nurse	6 months	14 years	No	35	F
VENCH024	Residential older adults	Senior carer	7 years	16 years	Yes	36	F
VENCH025	Residential behaviour and dementia	Service lead (MH nurse)	6 years	13 years	Yes	35	F
VENCH026	Residential dementia	Deputy manager	16 years	30 years	Yes	60	F
VENCH027	Residential dementia	Senior carer	20 years	25 years	No	54	F
VENCH028	Dementia nursing care	Compliance manager (MH nurse)	2 years	25 years	Yes	42	F
VENCH029	Residential dementia	Care support worker	6 months	2 years	No	26	F
VENCH030	Residential dementia and Parkinson’s	Night-care support worker	3 years	15 years	No	40	F
VENCH031	Nursing home	Care worker^a^	4 months	4 months	No	53	F

^a^This care worker stated they met the eligibility criteria but was subsequently found to have only had 4 months of experience working in a care home (eligibility criteria was 6 months). A decision was made to keep data from this participant. LD = learning disability. MH = mental health.

**Table 2.** Primary care clinician demographics

Participant ID	Role	Work setting	GP academic or special interest. Background and clinical management if not medical	Number of homes	Previous experience of research	Age, years	Sex
VENPC002	Advanced clinical practitioner	Care home assessment team in general practice	OT. Assesses and informs GP; does not prescribe	20	No	47	F
VENPC003	Older adult practitioner	Care home assessment team in general practice	Nurse. Assesses and informs GP; does not prescribe	20	No	48	F
VENPC004	GP partner	General practice	None	5–6	Yes	33	F
VENPC005	GP partner	General practice	PCN care home lead. Practice research lead	6	Yes	54	M
VENPC006	Older adult practitioner	Care home assessment team in general practice	Paramedic. Assesses, treats, and discusses with GP; does not prescribe	20	Yes	49	M
VENPC007	GP partner	General practice	PCN senior role	7	Yes	44	M
VENPC008	GP partner	General practice	Practice research lead	8	Yes	45	F
VENPC009	GP partner	General practice	Practice care home lead	2	No	45	F
VENPC010	Telemedicine team leader	Secondary and primary care, remote triage for residential homes	Manages clinical triage team of assessors; admits or refers to GP. Does not prescribe	200	No	37	F
VENPC011	Salaried GP	General practice	None	6	Yes	52	F
VENPC012	GP partner	General practice	PCN research lead	4	Yes	42	M

OT = occupational therapist. PCN = primary care network. GP = general practitioner.

**Figure 1.**
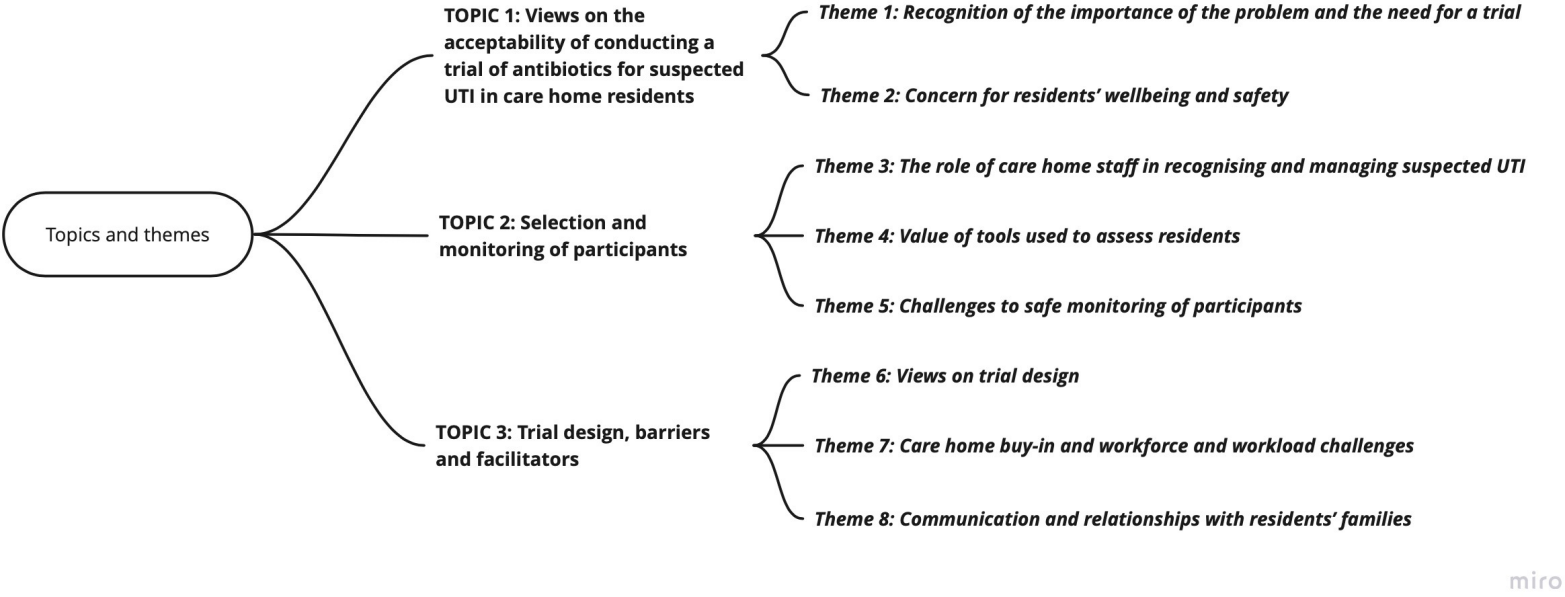
Diagram of topics and themes from the thematic analysis of care home staff and primary care clinician interviews. UTI = urinary tract infection

Eight themes, grouped under three topic areas that align with the aims of the study, were developed from the data (Figure 1).

### Topic 1: Views on the acceptability of conducting a trial of antibiotics for suspected UTI in care home residents

#### Theme 1: Recognition of the importance of the problem and the need for a trial

Carers reported that they recognised the importance of responsible antibiotic use. There was broad support for the trial among care home staff. They felt well-placed to undertake the trial in their care home, and felt confident that they would recognise subtle changes in their residents’ behaviour that might indicate the onset of infection.

Carers were also confident that residents and families would be supportive as long as the rationale was clearly communicated to them:

*'I think if it was clearly communicated* [to residents’ families] *and the rationale for why we're doing the study, I can't see why anyone wouldn't want to do that, yes.'* (VENCH025)

Among primary care clinicians, there was overwhelming support for the future trial in terms of its overall purpose and it was seen as potentially *'beneficial to future generations'* (VENPC010). Many primary care clinicians described the diagnostic challenge of UTIs, and recognised that they likely overprescribe because of diagnostic uncertainty and concerns about resident vulnerability. Many therefore expressed their desire for evidence to support withholding antibiotics on the initial observation of vague non-localising symptoms:

*'I think one of the biggest challenges … is that acute confused patient … a urinary infection can be the issue, but often, these patients have dementia, and other complex medical problems, that it can sometimes be a diagnostic conundrum.'* (VENPC008)

*'I think if there was more of a trust in withholding antibiotics. That would be helpful. I think this is what it’s* [the study] *all about. Holding fire* [from prescribing antibiotics] *… obviously the thing is with this group of patients, we want to keep them out of hospital*.*'* (VENPC006)

#### Theme 2: Concern for residents’ wellbeing and safety

Balanced against the value of the trial, both primary care clinicians and care home staff mentioned concerns about the safety of residents participating in a future trial. Some carers expressed concerns about the inclusion of ‘vulnerable’ residents and thought that this may put them at risk of rapid deterioration. These concerns could impact on participant selection and inclusion. However, most of those expressing concern thought that the trial would be acceptable if there was adequate safety netting in place.

*'The fear is probably that if somebody has an infection you wouldn't want them to go any time without having the treatment for it because of risk of sepsis or anything*.*'* (VENCH018)

Some carers also had concerns about the trial not meeting the necessary safeguarding, regulatory, and legislative requirements. Some carers were particularly concerned about including residents known to deteriorate rapidly, and also had concerns about consent and relationships with family members who did not want their family member to participate. Some thought those without capacity to consent should not be included, whereas others did not have this concern.

### Topic 2: Selection and monitoring of participants

#### Theme 3: The role of care home staff in recognising and managing suspected UTI

Both care home staff and primary care clinicians recognised the key role that care home staff play in identifying and acting on early features of suspected UTI. Care home staff reported spending a lot of time with the residents and felt they knew their residents very well. They felt ideally placed to notice subtle changes that might indicate the onset of symptoms:

*'Because we have a rapport with the residents, because we're living with them day in, day out, we would see something out of the norm in behaviour patterns*.*'* (VENCH016)

Primary care clinicians saw care home staff as having a key role in the management of residents’ clinical conditions, and reported that they highly valued carers for their knowledge and insight into their residents’ condition. However, some primary care clinicians described that there was an over-reliance on dipstick testing in care homes, as well as an expectation from care home staff for antibiotic prescribing, and felt they often did not see the ‘bigger picture’, which might reduce buy-in from care home staff and present a barrier to recruitment. Some primary care clinicians felt that managing expectations of care home staff, changing their beliefs and their behaviour, and therefore influencing change, was a key part of their role.

#### Theme 4: Value of tools used to assess residents

Many care home staff reported already using early warning scores (RESTORE2 or NEWS2) to assess residents’ physical observations and ‘soft’ signs (in the case of RESTORE2) when they appeared unwell. Staff were generally very positive about these tools, saying they empowered them and gave them confidence to recognise when it was safe to ‘watch and wait’ rather than escalate. They also indicated that they helped facilitate communication of the resident’s condition to other professionals, and felt such tools would be a good way of monitoring residents in a future trial:

*'Now with something like the RESTORE2, we've got a voice and a clinical side that we can produce and say, "Look, this is what’s happening." We didn't have that before. We were just carers*.*'* (VENCH019)

*'I think it* [RESTORE2] *should be used across the country, to be honest with you. I don't think you'll see any barriers.'* (VENCH028)

Similarly, primary care clinicians highlighted that a clear safety-netting process would be an important aspect of any future trial and there was general support for the use of such tools for detecting clinical deterioration of participants. All were aware of NEWS2, but most were not familiar with RESTORE2.

*'Most of our care homes give us the RESTORE2 result over the phone. They know what our expectation is, so they will do them* [RESTORE2 observations] *before they phone us. Through our education they are getting better at actually using it anyway and escalating if they think there’s a problem*.*'* (VENPC010)

Some primary care clinicians described a shift in the approach to the management of UTI since the introduction of the national Enhanced Health in Care Homes policy.^24^ Frailty teams, telemedicine, and paramedics provide additional support to some care homes, and may communicate with the GP after assessing the resident. However, not all homes have access to this, and this would need to be taken into consideration when recruiting care homes.

#### Theme 5: Challenges to safe monitoring of participants

Many care home staff emphasised the importance of training the care home workforce in the use of early warning scores, such as RESTORE2, if used in the study, and how this would need to be systematic and inclusive. There would need to be ongoing training for all staff who would need to use RESTORE2 in all participating care homes, including night and bank staff.

Some carers and primary care clinicians observed that it was important not to be too reliant on RESTORE2 and listen to their ‘gut instinct’. A small number of primary care clinicians also shared concerns about the value of RESTORE2 and its complexity. Some care home staff and primary care clinicians also raised concerns around the training requirements (including night and temporary staff), especially for care homes where it wasn’t currently used:

*'I think a robust education programme is going to be really beneficial because they are very, very protective of those residents.'* (VENPC010)

Care home teams felt it was important to have dedicated support from primary care, with GPs ready to engage with the trial. Some care home staff and primary care clinicians raised concerns about whether out-of-hours services would be able to provide adequate support for residents in a trial, as they would likely be unfamiliar with the resident and the nature of the trial. Some care home staff also raised concerns regarding the experience and knowledge of night and temporary staff working out of hours, and thought that a lack of continuity could lead to additional risks. Both groups expressed that clear communication and briefing and/or training with all staff members, including any additional support services covering out of hours (such as telemedicine and frailty teams), were paramount. Some thought the research team should be contactable at all times to ensure queries could be addressed promptly.

*'Sometimes* [policymakers] *don't necessarily take into account continuity of care. When I speak to a care home that I know, I know that carer, I trust how they assess patients … you've got that relationship where they trust me and I trust them ... That relationship is very different in an out-of-hours setting, where often it’s overnight, I've got to safeguard a lot more. So it’s a different consultation style.'* (VENPC012)

### Topic 3: Trial design, barriers, and facilitators

#### Theme 6: Views on trial design

Most carers understood the rationale for a placebo-controlled trial; however, some expressed concerns about the safety and legislation of such an approach. Those in support of the placebo-controlled design felt it would give better study outcomes and might be safer as all residents would be observed more closely. Most primary care clinicians were in favour of the blinded placebo-controlled approach, but some felt that the risks were too great, particularly in the out-of-hours period. Some felt that potential participants should be recruited in advance, before the onset of any symptoms.

Most particiants did not have specific recommendations for a primary outcome for the study. Primary care clinicians indicated that the trial should be designed to demonstrate whether or not withholding antibiotics in those with possible UTI with non-specific symptoms was a safe management strategy, and that there was not a significant increase in recovery time and serious outcomes. Some mentioned that it would be helpful for the findings to inform criteria that they could use to identify residents who would, and would not, benefit from antibiotics.

*’It would be actually looking at a patient's functional baseline. Then after 24/48 hours if the patient has received either a placebo or an antibiotic, are they back to their baseline? That would be the ultimate outcome for me.’* (VENPC010)

*’Decrease in antibiotic prescribing and no negative outcomes for patients.’* (VENPC007)

#### Theme 7: Care home buy-in and workforce and workload challenges

Engagement with care home staff was deemed critical for the success of a future trial, and how care home staff feel they are valued by other professionals contributes to their engagement. Care home managers valued their teams highly but some carers felt they were perceived as low value in the workforce:

*'*[During the COVID-19 pandemic] *we were referred to as "unskilled workers". We're not unskilled workers. We know our residents better than, sometimes, their families know them.'* (VENCH019)

However, they also reported an increase in sense of value during the COVID-19 pandemic, which was related to being asked to take on extra responsibilities such as monitoring residents’ observations.

Primary care clinicians reported highly valuing carers for their knowledge and insight into their residents. They described their relationship with the care home as one of trust, mutual respect, and support. Importantly, primary care clinicians felt that engagement and ‘buy-in’ from care home staff was critical for the success of any trial:

*'It’s important that care homes are involved in it, and I think that’s the key element to it. It’s those people in the care homes, and obviously calling us in a timely manner, recognising the signs because obviously they're the ones who are seeing them on a daily basis*.*'* (VENPC006)

Some care home staff were concerned about the feasibility of participating in a trial, especially given the current workload and staff shortages in care homes. However, other staff did not see the future trial as an extra burden.

*'You've got the time and cost barriers to the actual staff involved in the study, because if you're having to do extra observations under the RESTORE2, that all takes time. Some residential homes obviously don't use RESTORE2, so there’s the support and training on that.'* (VENCH024)

Many primary care clinicians also reported workload challenges, reporting that there are not enough GPs to deliver the work that is required already, which leads to pressure on the practice team. Consequently, residents are often not physically seen, and assessments are often carried out remotely:

*'There are issues with GP burnout, not enough GPs, GP recruitment, staff sickness, I think it honestly does impact across everything that we do.'* (VENPC008)

There were mixed opinions among primary care clinicians about whether the future trial itself would incur extra work for themselves or care home staff. Primary care clinicians were not concerned about being expected to assess residents in the trial who began to deteriorate as they felt it was their job to do that anyway.

#### Theme 8: Communication and relationships with residents’ families

Both groups highlighted that good relationships and communication with residents and their families would be essential for successful recruitment. Providing residents and families with clear information about the trial upfront (especially the rationale, requirements, and safety processes) would help build trust and improve recruitment, and staff would need to demonstrate reassurance and respect in their communication with families.

*'The importance of buy-in from relatives for the future study and for large care homes, real involvement from families would be needed*.*'* (VENPC007)

*'Before doing anything for the residents, any treatment or any new tablets or anything, we need to get consent from the family. We need to explain the pros and cons to the family, then they'll understand because we're looking after them and we always look the best for them. It’s an important thing that we need to get approval from the family as well*.*'* (VENCH029)

### Stakeholder meetings

Three stakeholder meetings were undertaken with residents and families, who were highly supportive of the planned trial overall, but emphasised the importance of clear communication and robust safety measures, including early warning scores.

## Discussion

### Summary

Care home staff and primary care clinicians (as well as residents and family members in the subsequent skateholder meetings) were broadly supportive of the proposed RCT. Prioritising the safety of residents was considered most important, and there was support for using early warning scores to monitor residents and identify clinical deterioration. Some care home staff and primary care clinicians seemed to lack equipoise about the potential value of a placebo-controlled design and were hesitant about the safety of this approach. However, they were more accepting when the safety systems and the value of a blinded trial were explained.

Care home staff with experience of using the RESTORE2 tool were very enthusiastic about its use and felt it empowered them, facilitating safer monitoring, decision making, and communication. Primary care clinicians were less familiar with RESTORE2, but all used the NEWS2 early warning score (itself a component of RESTORE2), and supported its use in a future trial. There was agreement that a robust training programme would be needed (to include all staff) around the use of any early warning tools.

Communication (with residents, families, and staff working both in and out of hours) was considered paramount to optimise recruitment. Carers were confident that families would be supportive if the rationale was clearly explained, and safety systems were robust. The perceived additional burden of the trial was seen as a barrier by some, and the use of temporary staff and the out-of-hours period were highlighted as potential risk areas.

### Strengths and limitations

This study’s strengths lie in the successful recruitment of an adequate and relevant sample of healthcare professionals who were able to understand the aims of the future trial, apply their experience to a hypothetical situation of being part of the trial, and envisage and articulate the facilitators and/or barriers to its delivery. A limitation of this study is that both care home staff and primary care clinicians self-selected as participants, and may have had different views from those who did not volunteer to participate. Additionally, a relatively high proportion of care home staff (56%) were in senior management roles, and their views might differ from more junior staff. It is not expected that the findings would be transferable to wider populations, but it is suggested that the findings provide useful insights for research teams in similar settings.

### Comparison with existing literature

It was encouraging to see support for the proposed future trial from both care home staff and primary care clinicians. Most trials on this topic have involved implementing guidelines and/or decision tools as part of antimicrobial stewardship education programmes.^25–27^ Such approaches may safely reduce antibiotic prescribing,^26,27^ but doubts remain over their sustainability and there are concerns of poor staff engagement in the long term, especially considering high staff turnover.^27^ Similar to the present study, a recent interview study with UK GPs also found that while the issue of antibiotic stewardship is well-acknowledged, GPs often justified antibiotic prescription for possible UTI with non-localising symptoms when residents were perceived to be at higher risk of deterioration, or if there was a preference for avoiding hospital admission, in which case antibiotics were seen as an alternative to doing ‘nothing’.^8^ This highlights the need for trials that explore the safety of withholding antibiotics in certain cases.

An important finding of the present study was support among both care home staff and primary care clinicians for the use of the RESTORE2 tool as a ‘safety net’ for recognising clinical deterioration. The use of early warning scores in care homes has increased considerably since the COVID-19 pandemic.^11,12^ Studies have demonstrated that clinicians value their use in remote monitoring and triage or management decisions, and that care home staff feel empowered by their use, improving communication with other healthcare professionals, and acting as an adjunct to their own intuition.^11,12^

Qualitative studies exploring research participation in care homes have suggested that ‘buy-in’ from care home staff is critical.^15,28,29^ The care home manager acts as a gatekeeper, and a good relationship with the research team is vital to facilitate introductions and gain the trust of staff, residents, and families.^15,28,29^ Additionally, junior care staff (who see residents more regularly) have a key relationship with residents and families, and their input is vital to optimise recruitment and maintain participation.^15,30^ The present study found that carers thought that most families would be supportive of the proposed trial as long as there was clear communication about the rationale. In previous studies, much of the hesitancy from staff and residents and/or families towards research has been explained by misconceptions about research, which may be considered ‘daunting’, and a lack of clear information.^15^

Efforts to reduce the extra burden on staff and facilitating effective communication between in- and out-of-hours teams, will also be vital to optimise uptake and engagement with any future trial. Care homes are busy and unpredictable settings, focusing foremost on resident care, and this presents a barrier to research engagement.^14,29^ Initiatives to help facilitate research include the National Institute for Health and Care Research ENRICH (Enabling Research in Care Homes) network, which aims to bring together researchers, care home staff, and residents, and support study design and delivery.

### Implications for research

There was broad support among participants for a proposed placebo-controlled RCT of antibiotics for possible UTI in care home residents with non-specific symptoms, as well as support for using the RESTORE2 tool to monitor participants in such a study. Future development of this trial will need to prioritise resident safety (especially in the out-of-hours period), effective communication, and minimising additional burden on staff to optimise recruitment.
